# The Effect of Exposure to a High-Fat Diet on MicroRNA Expression in the Liver of Blunt Snout Bream (*Megalobrama amblycephala*)

**DOI:** 10.1371/journal.pone.0096132

**Published:** 2014-05-02

**Authors:** Dingdong Zhang, Kangle Lu, Zaijie Dong, Guangzhen Jiang, Weina Xu, Wenbin Liu

**Affiliations:** 1 Key Laboratory of Aquatic Nutrition and Feed Science of Jiangsu Province, College of Animal Science and Technology, Nanjing Agricultural University, Nanjing, China; 2 Key Laboratory of Freshwater Fisheries and Germplasm Resources Utilization, Ministry of Agriculture, Freshwater Fisheries Research Center, Chinese Academy of Fishery Sciences, Wuxi, China; 3 Wuxi Fisheries College, Nanjing Agricultural University, Wuxi, China; Northeast Ohio Medical University, United States of America

## Abstract

Blunt snout bream (*Megalobrama amblycephala*) are susceptible to hepatic steatosis when maintained in modern intensive culture systems. The aim of this study was to investigate the potential roles of microRNAs (miRNAs) in diet-induced hepatic steatosis in this species. MiRNAs, small non-coding RNAs that regulate gene expression at the posttranscriptional level, are involved in diverse biological processes, including lipid metabolism. Deep sequencing of hepatic small RNA libraries from blunt snout bream fed normal-fat and high-fat diets identified 202 (193 known and 9 novel) miRNAs, of which 12 were differentially expressed between the normal-fat and high-fat diet groups. Quantitative stem-loop reverse transcriptase-polymerase chain reaction analyses confirmed the upregulation of miR-30c and miR-30e-3p and the downregulation of miR-145 and miR-15a-5p in high-fat diet-fed fish. Bioinformatics tools were used to predict the targets of these verified miRNAs and to explore potential downstream gene ontology biological process categories and Kyoto Encyclopedia of Genes and Genomes pathways. Six putative lipid metabolism-related target genes (fetuin-B, Cyp7a1, NADH dehydrogenase (ubiquinone) 1 beta subcomplex subunit 2, 3-oxoacid CoA transferase 1b, stearoyl-CoA desaturase, and fatty-acid synthase) were identified as having potential important roles in the development of diet-induced hepatic steatosis in blunt snout bream. The results presented here are a foundation for future studies of miRNA-controlled lipid metabolism regulatory networks in blunt snout bream.

## Introduction

Blunt snout bream (*Megalobrama amblycephala*) is a fish species of high economic value that is farmed in China's freshwater polyculture systems, as well as, increasingly, in other areas of Asia [1]. According to the latest FAO Fishery and Aquaculture Statistics yearbook, the total output of blunt snout bream in China reached 652,215 tons in 2010 [2]. Due to its herbivorous instinct and relatively low ratio of liver weight to body weight, blunt snout bream is highly susceptible to hepatic steatosis when fed a high-fat diet (HFD) for the purpose of protein sparing in an intensive culture system. Therefore, this species is a useful model to study the physiology of lipid metabolism and to compare it to that of other species such as zebrafish and medaka [3], [4].

Dietary fat comprises a group of water-soluble molecules that includes cholesterol and triglycerides. The liver synthesizes lipoproteins and, depending on the species, is more or less the epicentre of fatty-acid synthesis and lipid circulation. Accumulation of lipid droplets within hepatocytes results in hepatic steatosis, which may develop as a consequence of multiple dysfunctions, such as alterations in β-oxidation, very low-density lipoprotein secretion, and the activation of pathways involved in fatty-acid synthesis [5], [6]. Multiple hepatic transcription factors, including liver X receptors [7], retinoid X receptors [8], hepatocyte nuclear factors [9], peroxisome proliferator-activated receptors (PPARα, β and γ) [10], cAMP response element-binding protein [11], sterol regulatory element-binding proteins [12], and CCAAT/enhancer binding proteins [13], control gene networks that govern lipid synthesis, catabolism, storage, and secretion. Recently, microRNAs (miRNAs) have emerged as critical regulators of gene expression that control hepatic lipid metabolism at the posttranscriptional level [14].

MiRNAs are a distinct class of non-coding, single-stranded, 18–25 nucleotide (nt) RNA molecules that interact with the 3′-untranslated regions of target mRNAs and reduce protein synthesis by enhancing mRNA degradation and/or interfering with translation [15], [16]. Since the discovery of the first miRNA in *Caenorhabditis elegans* in 1993, thousands of mature miRNAs have been identified in a wide range of organisms, including animals, plants and viruses [17], [18]. Accumulating evidence suggests that miRNAs are involved in diverse biological processes, such as embryo development, cell differentiation, growth/proliferation, defense, apoptosis, signaling, and cancer [15], [19]. The involvement of miRNAs in energy metabolism was first indicated by a study of fruit fly (*Drosophila melanogaster*), which demonstrated an important role of miR-14 in fat metabolism at the whole-animal level [20]. Subsequent studies have revealed that miRNAs regulate the developmental and physiological processes of adipocyte differentiation, lipid metabolism, adipogenesis, glucose-stimulated insulin secretion, and HFD-induced weight gain [17], [20]–[22]. Recent advances in the understanding of lipid metabolism have revealed that a number of miRNAs, particularly miR-122 and miR-33, play major roles in regulating cholesterol and fatty-acid homeostasis in mice and may be promising targets for therapeutic interventions [23], [24].

Hepatic steatosis is a major obstacle to the sustainable development of the blunt snout bream industry in China. A comprehensive understanding of the mechanisms that lead to liver steatosis in fish remains elusive and the identification of target genes and pathways controlled by miRNAs is critical to understanding their function in lipid metabolism. High-throughput sequencing technologies can detect and quantify the expression levels of known and novel miRNAs. Here, hepatic small RNA libraries from blunt snout bream fed a normal-fat diet (NFD) or HFD for eight weeks were characterized. Deep sequencing and stem-loop reverse transcriptase-polymerase chain reaction (RT-PCR) validation were used to identify miRNAs that were differentially expressed between the two groups. Bioinformatics methods were used to predict targets of the verified miRNAs and to explore the potential downstream gene ontology categories and Kyoto Encyclopedia of Genes and Genomes (KEGG) pathways of the target genes. This analysis identified four miRNAs and six putative target genes that may be involved in hepatic lipid metabolism in blunt snout bream. Although a previous study characterized miRNAs involved in the growth of blunt snout bream by examining mixed pools of brain, pituitary, liver and muscle samples [25], to our knowledge, this study is the first report of miRNA profiling in the liver of this species. The identification of miRNAs and their target genes involved in liver steatosis will provide a better understanding of the biological processes of lipid metabolism and may identify novel targets for therapeutic intervention.

## Materials and Methods

### Ethics statement

All experimental protocols were approved by the Institutional Animal Care and Use Committee of Nanjing Agricultural University (Nanjing, China). To collect tissues, fish were anesthetized in well-aerated water with 0.01% tricaine methanesulfonate (Sigma, Saint Louis, USA) and were sacrificed according to the Guide for the Care and Use of Laboratory Animals of China.

### Experimental fish and feeding trial

Juvenile blunt snout bream collected from the Fish Hatchery of Wuhan (Hubei, China) were reared in a recirculating aquaculture system. After a week of acclimation, 200 healthy fish (weight: 20.24±0.11 g) were randomly divided into the NFD (5% fat diet) and HFD (15% fat diet) groups (n = 100 per group). Each 480 L tank housed 25 fish. The fish were hand-fed to apparent satiation three times a day (6:00–6:30, 12:00–12:30, and 18:00–18:30). Blunt snout bream were maintained in a controlled environment with a 12/12-hour light–dark cycle at 28°C. The formulation of the experimental diets and the environmental quality preferences were taken from established protocols [26], [27]. Each diet was tested in four replicate tanks and the trial lasted eight weeks.

### Sample collection and RNA extraction

After eight weeks of rearing, liver tissues were removed from the fish, immediately frozen in liquid nitrogen, and then stored at -80°C until use. For miRNA sequencing, a total of 16 fish (validated by oil red O staining; see below) from two groups were selected (one male and one female from each of eight tanks). Total RNA was extracted using the mirVana microRNA Isolation Kit (Ambion, Austin, USA), according to the manufacturer's instructions. The quality and quantity of the total RNA were determined using an Agilent 2100 Bioanalyzer (Agilent, CA, USA). RNA samples with a RNA integrity number >8.0 were processed for sequencing. 8 RNA samples from NFD group with equivalent RNA concentration and 8 RNA samples from HFD group with equivalent RNA concentration were pooled together for sequencing, respectively.

### Oil red O staining

After washing three times with phosphate-buffered saline, sliced liver samples were fixed with 10% formalin in phosphate buffer for 1 h at room temperature. The samples were then washed with phosphate-buffered saline and stained with a filtered oil red O (Sigma-Aldrich) solution (0.5 g in 100 ml isopropyl alcohol) for 15 min at room temperature. After staining, the samples were rinsed twice with distilled water for 15 min. The sections were also counterstained with Mayer's hematoxylin to visualize the nuclei [4].

### Small RNA library preparation and sequencing

Small RNAs (16–30 nt) were isolated from the total RNA samples by size fractionation using 15% denaturing polyacrylamide gel electrophoresis. Proprietary adaptors (Illumina, San Diego, USA) were then ligated to the 5′ and 3′ ends of the small RNAs and reverse transcription was performed according to the Illumina protocol. The generated small cDNA libraries were amplified by PCR using primers complementary to the adaptor sequences. The cDNA libraries were then deep sequenced using the HiSeq2000 system (Illumina, San Diego,USA) at Beijing Genomics Institute (Shenzhen, China), according to the manufacturer's instructions. All small RNA and RNASeq data are available in the NCBI SRA (Sequence Read Archive) under accessions SRX494382 and SRX494377 respectively.

### Sequence analysis and miRNA identification

An initial filtering step was performed to exclude poor quality reads, 3′ adaptor reads, reads with 5′ adaptor contaminants, and reads shorter than 18 nt. The remaining sequences were mapped to the zebrafish (*Danio rerio*) genome using the SOAP program (http://soap.genomics.org.cn) with a tolerance of one mismatch. The matched sequences were blasted against the Rfam (http://rfam.sanger.ac.uk/) and NCBI GenBank (http://www.ncbi.nlm.nih.gov/genbank/) databases to filter out rRNAs, tRNAs, snRNAs and snoRNAs. After being classified into different categories based on sequence similarity, the remaining reads were aligned to the miRBase version 20 database (http://www.mirbase.org/) to identify conserved miRNAs [28]. The *M. amblycephala* genome is currently unavailable; therefore, MIREAP (http://sourceforge.net/projects/mireap) was used to predict the secondary structures, Dicer enzyme cleavage sites and minimum free energies of the unannotated small RNAs that mapped to the zebrafish genome sequence. Small RNAs were considered candidate novel miRNAs if they fulfilled the following MIREAP criteria: specific length between 18 and 26 nt, specific length of the miRNA reference sequence between 20 and 24 nt, minimal depth of the Drosha/Dicer cutting site equal to 3 nt, maximum copy number of miRNAs on reference equal to 20 nt, free energy allowed for a miRNA precursor ≤−18 kcal/mol, space between miRNA and miRNA* ≤35 nt, base pairs of miRNA and miRNA* ≥14 nt, bulge of miRNA and miRNA* ≤4 nt, asymmetry of the miRNA/miRNA* duplex ≤5 nt, and flank sequence length of the miRNA precursor equal to 10 nt [29].

### Differential expression analysis of the sequencing data

To compare the expression levels of miRNAs in the cDNA libraries prepared from the NFD and HFD groups, the sequencing data were normalized as follows: 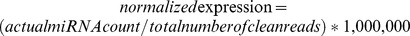. If the normalized expression of a given miRNA was zero, its expression value was set as 0.01. In addition, miRNAs with normalized expression values <1 in both samples were removed from the differential expression analysis. The fold change between miRNA expression levels in the NFD and HFD groups was determined as follows: 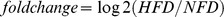. *P*-values were generated from the normalized expression values as shown below [29], where *N_1_* and *N_2_* represent the total number of clean reads in the HFD and NFD libraries, respectively, and *x* and *y* represent the normalized expression level of a given miRNA in the HFD and NFD libraries, respectively:
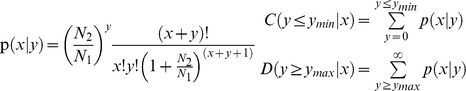



### Quantitative real-time PCR analysis

Reverse transcription of miRNAs was performed using miRNA-specific stem-loop primers and the PrimeScript RT Reagent Kit (Takara Bio, Dalian, China). Each 20 µl reaction contained 1 µl of PrimeScript RT Enzyme Mix I, 4 µl of 5× PrimeScript Buffer, 6 µl of nuclease-free water, 5 µl of RNA template, and 4 µl of stem-loop primer (Tables S1 and S2). Reverse transcription was performed by incubating the reactions at 16°C for 30 min, 42°C for 30 min, and then 85°C for 5 min. Real-time PCR amplification was performed using SYBR Premix EX Taq II Kit (Takara Bio, Dalian, China). Each 25 µl reaction included 1.3 µl of cDNA template, 12.5 µl of SYBR Premix EX Taq II, 1 µl of miRNA-specific forward primer (10 µM), 1 µl of universal reverse primer (10 µM), and 9.2 µl of RNase-free water. Thermal cycling was performed on a 7900HT Fast Real-Time PCR System (Applied Biosystems, Foster, USA) as follows: 95°C for 10 min, followed by 40 cycles of 95°C for 30 s, 60°C for 30 s, and 72°C for 45 s. A melting curve program was performed after amplification. The data were analyzed via the comparative *C*_T_ method [△C_T_ = C_T_(miRNA) – C_T_(Rpl13a)], using Rpl13a expression as the endogenous reference [30], [31].

Relative miRNA target genes expression was determined using PrimeScript RT Master Mix Kit and SYBR Premix Ex Taq II Kit (Takara Bio, Dalian, China). Real-time PCR protocol was initiated at 95°C for 10 min, followed by 40 cycles of a two-step amplification programme (15 s at 95°C; 40 s at 60–62°C), according to the primer set used (Table S3). Melting curves were systematically monitored at the end of the last amplification cycle to confirm the specificity of the amplification reaction. The data were analyzed via the comparative *C*_T_ method [△C_T_ = C_T_(mRNA) – C_T_(ACTB)], using ACTB expression as the endogenous reference.

### Prediction and analysis of miRNA target genes

Target genes of differentially expressed miRNAs were identified using the RNAhybrid (http://bibiserv.techfak.uni-bielefeld.de/rnahybrid) and TargetScan (http://www.targetscan.org/) prediction packages. The criteria used for target prediction were as follows: (i) no more than four mismatches between the small RNA and the target (G-U bases counted as 0.5 mismatches), (ii) no more than two adjacent mismatches in the miRNA/target duplex, (iii) no adjacent mismatches in positions 2–12 of the miRNA/target duplex (5′ end of the miRNA), (iv) no mismatches in positions 10–11 of the miRNA/target duplex, (v) no more than 2.5 mismatches in positions 1–12 of the miRNA/target duplex (5′ end of the miRNA), and (vi) minimum free energy (MFE) of the miRNA/target duplex ≥75% of the MFE of the miRNA bound to its perfect complement. Lipid metabolism-related target genes that were identified by both RNAhybrid and TargetScan, and that were present in the results of our *M. amblycephala* hepatic comparative transcriptome sequencing analysis (data not shown), were considered for further investigation. Functions that were significantly associated with the predicted target genes of the miRNAs were determined via a GO (http://www.geneontology.org) biological process analysis and a KEGG pathway analysis (http://www.genome.jp/kegg/pathway.html).

## Results and Discussion

### Hepatic accumulation of lipids in HFD-fed and NFD-fed blunt snout bream

**Figure 1:**
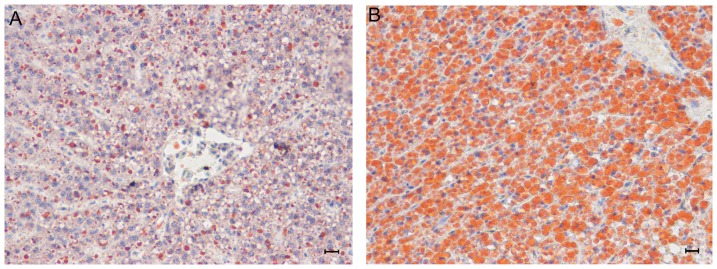
Hepatic lipid accumulation in blunt snout bream fed a normal-fat diet (NFD) or high-fat diet (HFD). Photomicrographs (×400) of liver tissue samples from blunt snout bream fed a NFD (A) or HFD (B) for eight weeks. Scale bar, 50 µm.

Exposure to a HFD can be used to induce hepatic steatosis in animal models [26]. To examine lipid metabolism and identify miRNAs related to hepatic steatosis, blunt snout bream were fed a HFD or NFD for eight weeks. Oil red O staining of liver tissue samples revealed the presence of severe hepatic lipid accumulation in HFD-fed fish but not NFD-fed fish (Figs. 1A and 1B).

### Small RNA profiles in HFD-fed and NFD-fed blunt snout bream

To identify miRNAs involved in lipid metabolism in blunt snout bream, independent hepatic small RNA libraries were generated from the NFD and HFD groups and then sequenced using the Illumina Hiseq2000 platform. A total of 13,932,306 and 12,928,706 raw reads were generated from the NFD and HFD libraries, respectively. After filtering out the adaptor sequences, low quality sequences and sequences smaller than 18 nt, these numbers were reduced to 13,809,426 and 12,854,691 mappable small RNA sequences in the NFD and HFD libraries, respectively (Table S4). The size distributions of the reads in the two libraries were similar; in both libraries, most (>94%) of the small RNAs were 21–23 nt in length. Small RNAs of 22 nt, which is the typical length of Dicer-derived products, accounted for 61.93% and 65.29% of the total sequence reads in the NFD and HFD libraries, respectively (Fig. S1). These results are consistent with the typical length distributions of small RNAs in other fish and animal species, such as common carp [32], channel catfish [19], goat [33], and honey bee [34].

The rRNA, tRNA, snRNA, snoRNA, scRNA, repeat DNA, exon_antisense, exon_sense, intron_antisense, and intron_sense sequences accounted for 1.45% and 0.70% of the total sequence reads, and 17.21% and 19.37% of the unique sequence reads, in the NFD and HFD small RNA libraries, respectively (Table S4). A large number of unique reads (81.45% in the NFD library and 78.85% in the HFD library) were attributed to unannotated sequences. Conserved miRNAs accounted for 86.35% and 91.11% of the total sequence reads, and 1.34% and 1.78% of the unique sequence reads, in the NFD and HFD libraries, respectively. These results suggest that, like other tissues, the liver of blunt snout bream contains large numbers of various types of small non-coding RNAs, which may contribute to the regulation of gene expression.

Due to the lack of whole genome data for *M. amblycephala*, the selected small RNA sequences were mapped to the genome sequence of zebrafish (*D. rerio*), which is evolutionarily close to *M. amblycephala* [25]. For the NFD group, 12,075,022 reads (87.44%) representing 18,565 unique small RNAs were mapped to the reference genome. Similarly, for the HFD group, 11,756,623 reads (91.46%) representing 15,008 unique small RNAs were mapped to the reference genome (Table S4).

### Identification of conserved miRNAs and classification of biological processes

**Figure 2:**
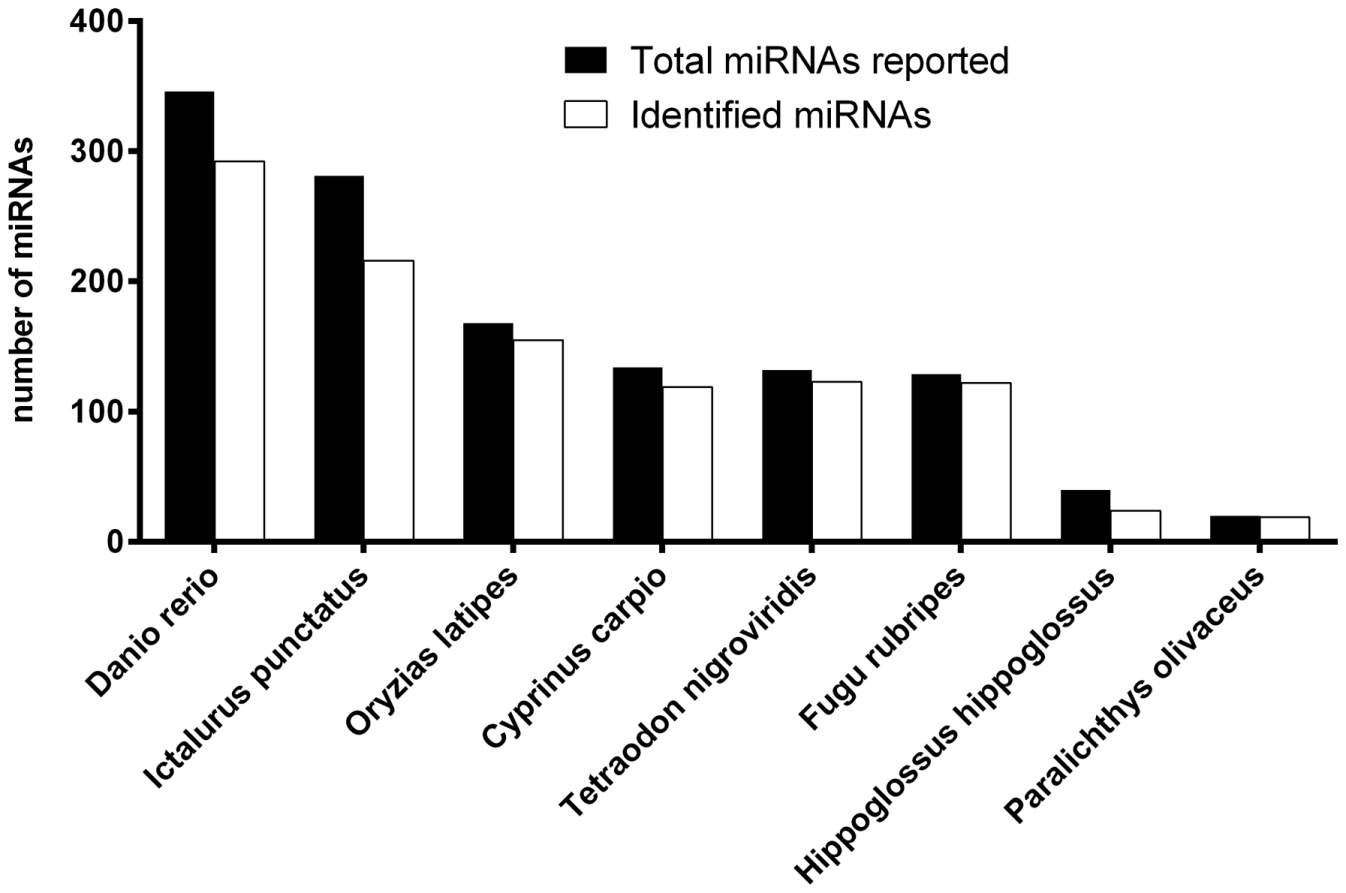
Overlap between the miRNA sequences identified in blunt snout and those from other fish species. The graph shows the numbers of miRNAs from the indicated species (listed in the miRBase version 20 database) that correspond to those identified in blunt snout bream livers.

To identify conserved blunt snout bream miRNAs, the sequences of the miRNAs in the libraries prepared from the NFD and HFD groups were compared with those of the 346 precursor miRNAs and 255 mature miRNAs from *D. rerio* listed in miRBase version 20.0. This analysis identified 189 and 175 conserved miRNAs in the NFD and HFD libraries, respectively (Table S5). After grouping identical sequences, a total of 193 mature miRNAs were obtained from these two libraries. In addition to *D. rerio*, these miRNAs could be mapped to a large proportion of the miRNA precursors from a number of other fish species listed in miRBase version 20, including channel catfish (*Ictalurus punctatus*), medaka (*Oryzias latipes*), common carp (*Cyprinus carpio*), spotted green puffer (*Tetraodon nigroviridis*), Japanese pufferfish (*Fugu rubripes*), atlantic halibut (*Hippoglossus hippoglossus*), and Japanese flounder (*Paralichthys olivaceus*) (Fig. 2).

**Figure 3:**
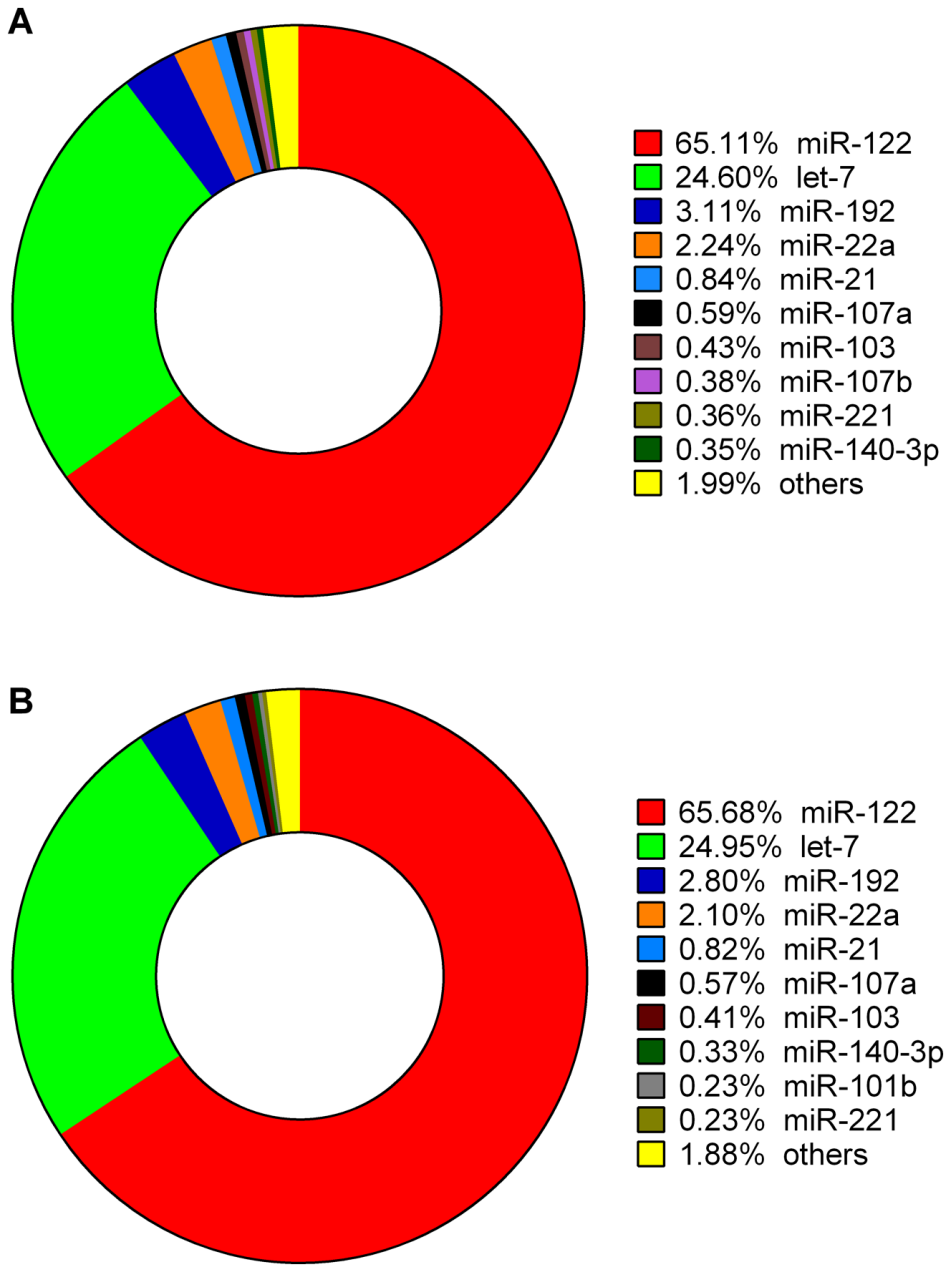
The ten most abundant miRNAs in the normal-fat diet (NFD) and high-fat diet (HFD) groups. The charts show the frequencies of the known miRNAs identified in the NFD (A) and HFD (B) groups.

The sequencing frequency of a miRNA generally reflects its abundance within a sample. The ten most abundant miRNAs in the NFD library were miR-122, let-7, miR-192, miR-22a, miR-21, miR-107a, miR-103, miR-107b, miR-221 and miR-140-3p, accounting for 98.01% of the total reads mapped to miRBase (Fig. 3A). Nine of these miRNAs were also among the ten most abundant miRNAs identified in the HFD library (miR-122, let-7, miR-192, miR-22a, miR-21, miR-107a, miR-103, miR-140-3p, miR-101b, and miR-221), which accounted for 98.12% of the total reads mapped to miRBase (Fig. 3B). The five most abundant miRNAs identified in both groups (miR-122, let-7, miR-192, miR-22a and miR-21) are also expressed at high levels in human liver [35], suggesting that they play fundamental roles in hepatic processes.

As described for other fish species and mammalian species [19], [36], miR-122 and let-7 were the most abundant miRNAs identified in *M. amblycephala* liver, and, together, these miRNAs accounted for 89.71% and 90.63% of the known miRNAs in the NFD and HFD groups, respectively. MiR-122 was one of the first reported examples of a tissue-specific miRNA in mammals. This miRNA is expressed at high levels in the liver and constitutes 70% of the total miRNA pool in mouse liver [36], [37]. Similarly, miR-122 accounted for 65.11% and 65.68% of the total reads mapped to miRBase in the NFD and HFD libraries, respectively (Figs. 3A and 3B). MiR-122 is highly conserved from humans to frogs, suggesting that it plays an important role in liver function and has hence been subjected to selection pressure throughout evolution [35], [36], [38], [39]. Ten members of the let-7 family, including let-7a, let-7b, let-7c, let-7d, let-7e, let-7f, let-7g, let-7h, let-7i and let-7j, were present at high frequencies in the *M. amblycephala* liver tissue. Notably, the numbers of counts of the mature sequences of these let-7 family members were similar between the NFD and HFD libraries (Table S5). Because let-7 family members are expressed ubiquitously, they were not examined in more detail here. In both libraries, let-7a was the most abundant let-7 family member identified. In total, let-7 miRNAs accounted for 24.60% and 24.95% of the known miRNAs in the NFD and HFD groups, respectively (Fig. 3). These results are similar to those reported in a previous study of miRNAs in different common carp tissues [32]. Roush et al. [40] also demonstrated that the let-7 family is expressed at high levels and is conserved across animal species, including human, flies, and worms, and plants. In general, these findings suggest that let-7 miRNAs are important regulators of fundamental biological processes [41], [42].

### Identification of novel miRNAs

Next, the unannotated small RNAs from the *M. amblycephala* libraries that mapped to the *D. rerio* genome were analyzed further. MIREAP software was used to predict novel miRNAs based on secondary structure, Dicer enzyme cleavage sites, and the MFE. To minimize noise, sequencing tags with fewer than five reads were excluded [43], [44]. Nine candidates with the typical miRNA stem-loop secondary structure, which forms the Dicer enzyme cleavage site, were identified (Table S6 and Fig. S2). The MFEs of the predicted pre-miRNAs ranged from −19.20 kcal/mol to −36.00 kcal/mol (mean: −28.83 kcal/mol). Seven of these miRNA candidates were identified in the NFD library, five were identified in the HFD library, and three were identified in both libraries (Table S6). To validate the sequencing data and bioinformatics predictions, the expression levels of the three candidate novel miRNAs that existed in both libraries (novel_mir-2, novel_mir-4, and novel_mir-7), were validated by stem-loop RT-PCR. The results of these analyses confirmed the existenceapp:ds:accuracy of these three novel miRNAs in blunt snout bream (Fig. S3 and Table S1).

### Differential expression of conserved miRNAs

**Figure 4:**
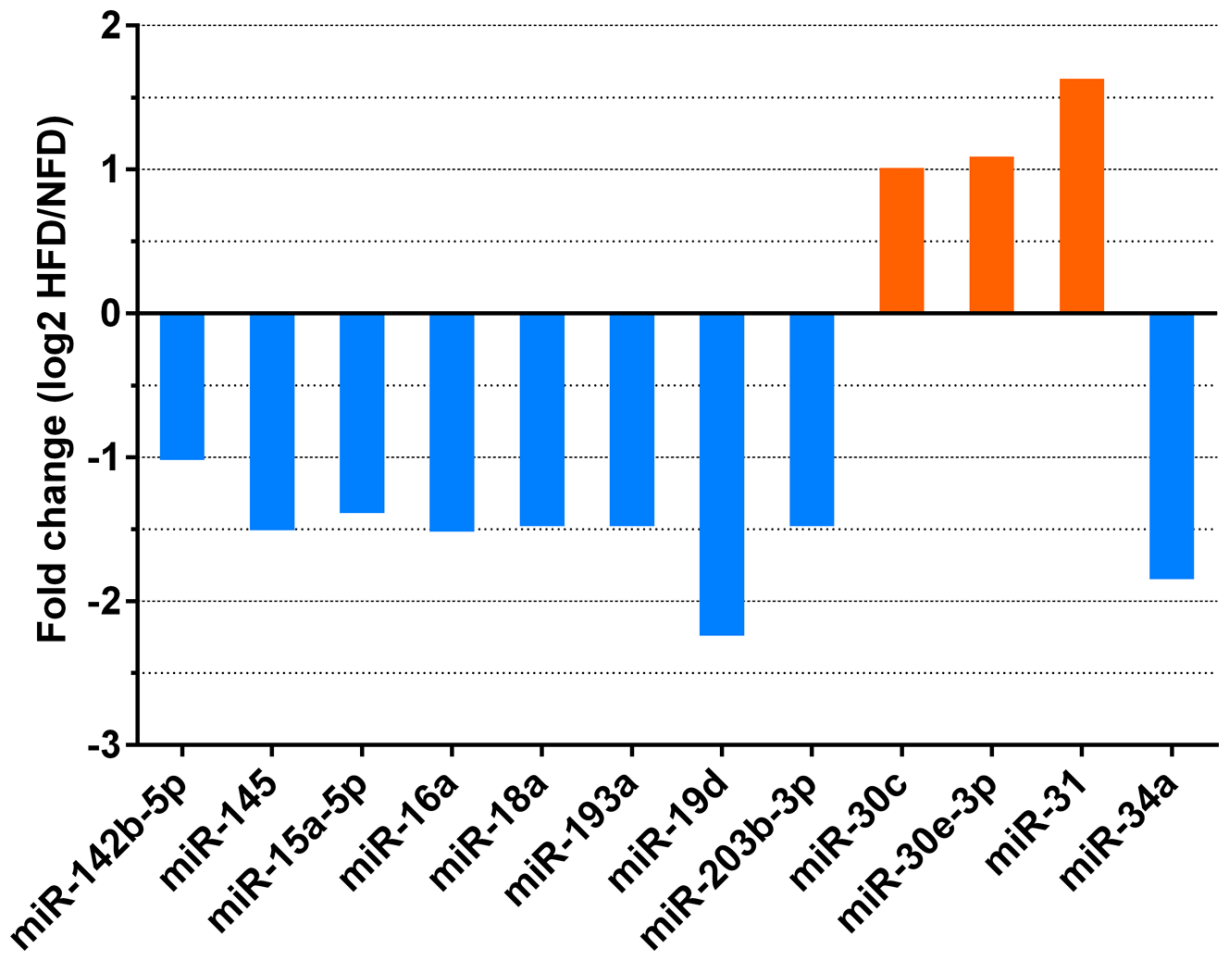
Fold changes of miRNAs that were differentially expressed between the normal-fat diet (NFD) and high-fat diet (HFD) groups. Fold changes were calculated as log2 (HFD/NFD).

**Figure 5:**
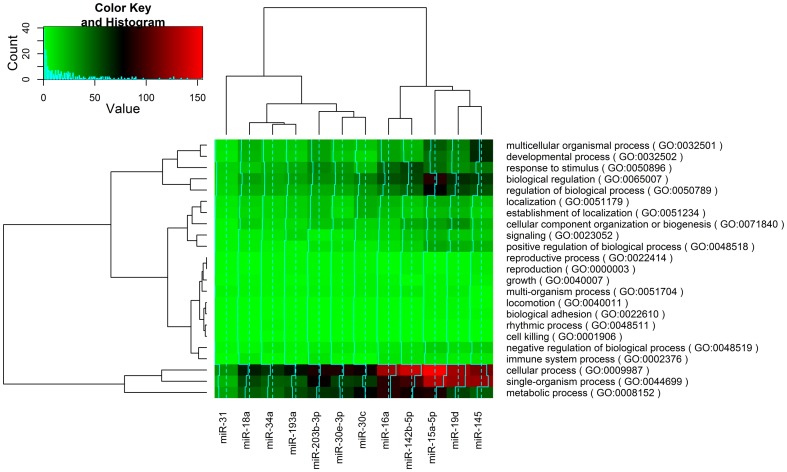
Cluster analysis of the differentially expressed miRNAs. A heat map was constructed using an R script based on the 23 biological processes of the 6,001 miRNA target genes identified by a gene ontology analysis. The coloring indicates the numbers of potential targets of the miRNAs.

After removal of sequencing tags with fewer than five read counts, 12 conserved miRNAs were identified as differentially expressed between liver tissues from HFD-fed and NFD-fed fish (Fig. 4). Compared with their expression levels in the NFD group, three miRNAs (miR-30c, miR-30e-3p, and miR-31) were upregulated and nine miRNAs (miR-142b-5p, miR-145, miR-15a-5p, miR-16a, miR-18a, miR-193a, miR-19d, miR-203b-3p, miR-34a) were downregulated in the HFD group. Using homologous sequences in the zebrafish genome, putative targets of these miRNAs were predicted using RNAhybrid software. The identified target genes were also subjected to a GO analysis, which classifies miRNA-gene regulatory networks on the basis of biological processes [45]. The GO analysis identified 6,001 predicted targets that were classified into 23 biological processes. A heat map analysis indicated that the functions of these miRNAs clustered into four groups and were associated mainly with metabolic, single-organism and cellular processes (Fig. 5).

**Figure 6:**
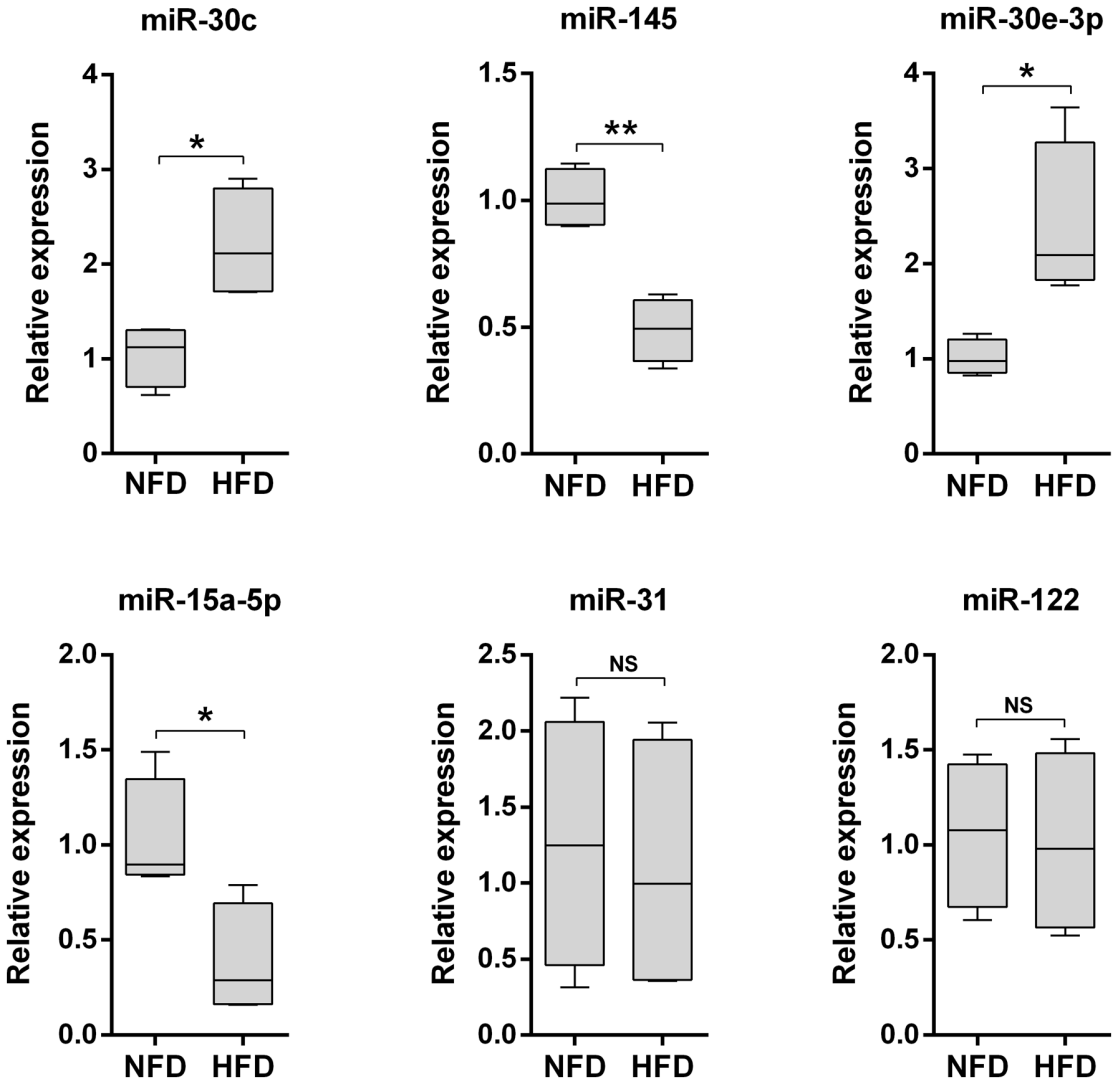
Quantitative reverse transcriptase-polymerase chain reaction validation of mature miRNA expression levels in the normal-fat diet (NFD) and high-fat diet (HFD) groups. Box and whisker plots of the expression levels of the indicated miRNAs in the NFD and HFD groups (n = 10 replicates per group). The expression level of each miRNA was normalized to that of Rpl13a. The upper and lower limits of the box are the first and third quartiles, and the horizontal line inside the box is the second quartile (median). *P<0.05 and **P<0.01 by unpaired Student's *t*-tests. NS, no significant difference.

Next, stem-loop quantitative RT-PCR (qRT-PCR) was used to validate the differential expression levels of miR-30c, miR-145, miR-30e-3p, miR-15a-5p, and miR-31 (Table S2), which were all detected at relatively high copy numbers in the NFD and HFD samples (Table S5). The qRT-PCR analysis confirmed that the expression levels of miR-145 and miR-15a-5p in the HFD group were significantly lower than those in the NFD group (Fig. 6). The analysis also confirmed that the expression levels of miR-30c and miR-30e-3p in the HFD group were significantly higher than those in the NFD group, although it did not confirm the upregulation of miR-31 expression in the HFD group (Fig. 6). In general, analysis of the number of reads generated by high-throughput sequencing is a reliable method of quantifying miRNA expression [44], [46], [47]; however, Cristino et al. [44] suggested that solexa sequencing data with fewer than 100 reads are unreliable for quantitative analyses. Of the five differentially expressed miRNAs examined by qRT-PCR here, miR-31 was the only one that produced fewer than 100 sequencing reads (48 reads in the NFD library and 138 reads in the HFD library) (Table S5), which may explain the discrepancy between the results of the deep sequencing and qRT-PCR analyses of this miRNA.

Previous studies have demonstrated that miR-30c, miR-30e-3p, miR-145, and miR-15a-5p play important roles in lipid metabolism. In a recent study by Soh et al. [48], hepatic over-expression of miR-30c attenuated atherosclerosis in Apoe^-/-^ mice and reduced hyperlipidemia in western diet-fed mice by inhibiting lipid synthesis and the secretion of triglyceride-rich ApoB-containing lipoproteins. Moreover, in the same study, inhibition of hepatic miR-30c promoted hyperlipidemia and atherosclerosis. MiR-30e is expressed at high levels in subcutaneous adipose tissue and may be involved in the association between adipose tissue dysfunction and the development of obesity-related disorders such as noninsulin-dependent diabetes mellitus [49], whereas MiR-145 expression is downregulated in nonalcoholic steatohepatitis [50]. MiR-15a may promote adipogenesis by inhibiting delta-like 1 homolog [51].

Previous studies have shown that liver-specific miR-122 is a critical regulator of cholesterol and fatty-acid metabolism in mice, suggesting that it may be an attractive therapeutic target for metabolic disease [36]; therefore, the expression levels of this miRNA in the NFD and HFD samples were also determined by qRT-PCR. Consistent with the sequencing results, there was no significant difference between the miR-122 expression levels in the NFD and HFD groups (Fig. 6).

### MiRNA target prediction and qPCR validation

**Figure 7:**
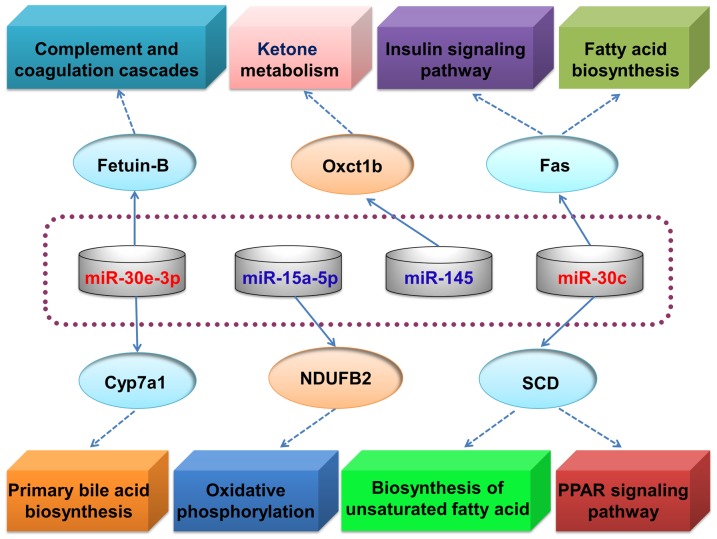
Predicted lipid metabolism-related miRNA target genes that were regulated by exposure to a high-fat diet. Schematic representation of potential determinants of the pathogenesis of hepatic steatosis in blunt snout bream.

Bioinformatics approaches that are based on the high degree of homology between miRNAs and their target genes are useful and effective tools for identifying miRNA target genes [52]. Popular miRNA target prediction packages, such as TargetScan [53], [54], DIANA-microT [55], RNAhybrid [56], PicTar [57], and miRanda [58], typically produce large numbers of potential targets for each miRNA. Therefore, to refine and improve the target gene prediction results and to limit the validation requirements, lipid metabolism-related target genes that were identified by both RNAhybrid and TargetScan, and that were present in the results of our *M. amblycephala* hepatic comparative transcriptome sequencing analysis (data not shown), were considered for further investigation. Using this approach, six lipid metabolism-related genes were identified as potential targets of four of the miRNAs that were differentially expressed between the NFD and HFD groups (Fig. 7).

Fetuin-B and Cyp7a1 (cytochrome P450, family 7, subfamily A, polypeptide 1a) were identified as potential targets of miR-30e-3p, which was expressed at higher levels in the HFD group than the NFD group. In addition, stearoyl-CoA desaturase (SCD) and fatty-acid synthase (Fas) were identified as potential targets of miR-30c, which was also expressed at higher levels in the HFD group than the NFD group. Fetuin-B mRNA levels are reduced during the acute phase of experimentally induced inflammation in rats [59] and downregulation of fetuin-B has been linked to impaired fatty-acid metabolism in liver cells [60]. Cyp7a1 is the initial and rate-determining enzyme in the classic and alternative pathways of bile acid synthesis from cholesterol, which is a major pathway for removal of cholesterol from the body [61]. Fas is a key enzyme involved in hepatic lipogenesis that is responsible for the synthesis of long-chain saturated fatty acids [62]; two important transcription factors, upstream stimulatory factor and sterol regulatory element-binding protein-1c, which are induced by feeding or insulin, play a dominant and cooperative role in regulating transcription of the gene encoding Fas [63]. SCD catalyzes the conversion of saturated fatty acids to Δ-9 monounsaturated fatty acids, which are important precursors for the formation of complex lipids such as phospholipids, triglycerides, cholesterol esters, wax esters, and diacylglycerols [64]. SCD is positively regulated by PPARγ, which is associated with lipid metabolism [65]. Therefore, in blunt snout bream fed a HFD, miR-30e-3p and miR-30c may disrupt lipid metabolism by attenuating the expression levels of fetuin-B, Cyp7a1, SCD and Fas.

NADH dehydrogenase (ubiquinone) 1 beta subcomplex subunit 2 (NDUFB2) and 3-oxoacid CoA transferase 1b (Oxct1b) were identified as potential targets of miR-15a-5p and miR-145, respectively. Both of these miRNAs were expressed at lower levels in the HFD group than the NFD group, suggesting that NDUFB2 and Oxct1b may be determinants of the pathogenesis of hepatic steatosis in blunt snout bream. Acetyl-CoA formed in the liver is converted into ketone bodies that are utilized by extrahepatic tissues. Oxct1 is a mitochondrial matrix enzyme that is essential for ketone body oxidation [66]. Buchner et al. [67] reported that NDUFB2, which is involved in oxidative phosphorylation, is over-expressed in the liver of 6C1 mice susceptible to diet-induced obesity.

**Figure 8:**
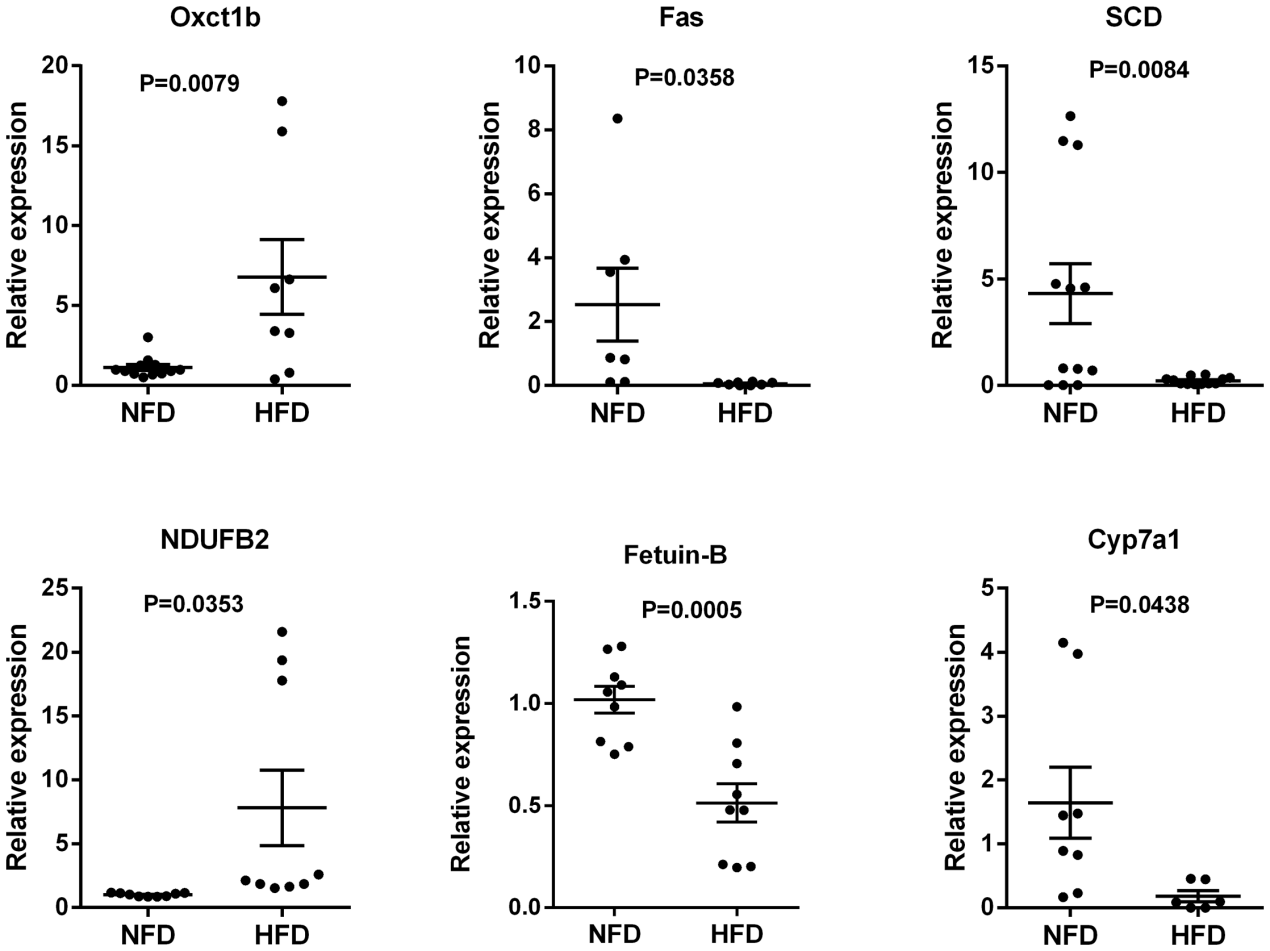
Quantitative reverse transcriptase-polymerase chain reaction validation of miRNA target gene expression levels in the normal-fat diet (NFD) and high-fat diet (HFD) groups. The expression levels of the indicated lipid metabolism-related genes in the NFD and HFD groups (n = 6–12 replicates per group) were normalized to that of β-actin (ACTB). The short horizontal lines represent the first and third quartiles, and the long horizontal line indicates the second quartile (median). Statistical analyses were performed using unpaired Student's *t*-tests. NS, no significant difference.

Partial or complete *M. amblycephala* cDNA sequences were not available for the six lipid metabolism-related miRNA target genes described above; therefore, the cDNA sequences of these genes were cloned, Sanger sequenced, and submitted to GenBank (Table S3). Next, qRT-PCR analyses were used to validate the bioinformatics predictions. Consistent with the expected inhibitory effects of miRNAs on their target genes, the expression levels of the mRNAs encoding fetuin-B, Cyp7a1, SCD, and Fas were significantly lower in the HFD group than the NFD group and the expression levels of the mRNAs encoding Oxct1b and NDUFB2 were significantly higher in the HFD group than the NFD group (Fig. 8).

## Conclusions

To our knowledge, this study is the first description of the miRNA expression profile of blunt snout bream liver. The effects of exposure of the fish to a HFD on the expression levels of miRNAs and their predicted lipid metabolism-related target genes were characterized. In addition, the potential downstream GO categories and KEGG pathways of the target genes were determined. The results suggest that four verified miRNAs and six putative target genes play important roles in hepatic steatosis in blunt snout bream. The results presented here are a foundation for future studies aimed at identifying potential pharmaceutical and nutritional targets in blunt snout bream. Additional *in vivo* and *in vitro* investigations are required to gain a complete understanding of the association between miRNA profiles, target genes, and the pathogenesis of hepatic steatosis in this species.

## Supporting Information

Figure S1 **Size distributions of small RNAs identified in the normal-fat diet and high-fat diet libraries.**
(TIF)

Figure S2 **Sequences and structures of the three novel miRNAs identified in the normal-fat diet and high-fat diet groups.** Precursor sequences, predicted stem-loop structures and minimum free energies (△G in kcal/mol) of novel_mir-2 (A), novel_mir-4 (B), and novel_mir-7 (C). The sequences of the corresponding mature miRNAs are indicated by red rectangles.
(TIF)

Figure S3 **Stem-loop reverse transcriptase-polymerase chain reaction confirmation of the expression of three novel miRNAs in blunt snout bream.** M, DNA marker; Neg, negative control (no DNA/RNA); lane 1, novel_mir-2; lane 2, novel_mir-4; lane 3, novel_mir-7; Pos, positive control (endogenous miR-122).
(TIF)

Table S1 **Details of the primers used for stem-loop reverse transcriptase-polymerase chain reaction validation of three novel miRNA candidates.**
(XLS)

Table S2 **Details of the primers used for stem-loop reverse transcriptase-polymerase chain reaction validation of conserved miRNAs.**
(XLS)

Table S3 **Details of the GenBank accession numbers and primers used to amplify the miRNA target genes.**
(XLS)

Table S4 **The sequencing counts originating from known RNA classes in the normal-fat diet and high-fat diet libraries.**
(XLS)

Table S5 **List of the conserved miRNAs identified in the normal-fat diet (NFD) and high-fat diet (HFD) libraries.**
(XLS)

Table S6 **Summary of the novel miRNAs identified in the normal-fat diet and high-fat diet libraries.**
(XLS)
