# Supplementary figures and images for: The Effect of Exposure to a High-Fat Diet on MicroRNA Expression in the Liver of Blunt Snout Bream (Megalobrama amblycephala)
Source: PLoS One. 2014 May 2;9(5):e96132. doi: 10.1371/journal.pone.0096132 (PMC4008502; doi:10.1371/journal.pone.0096132)

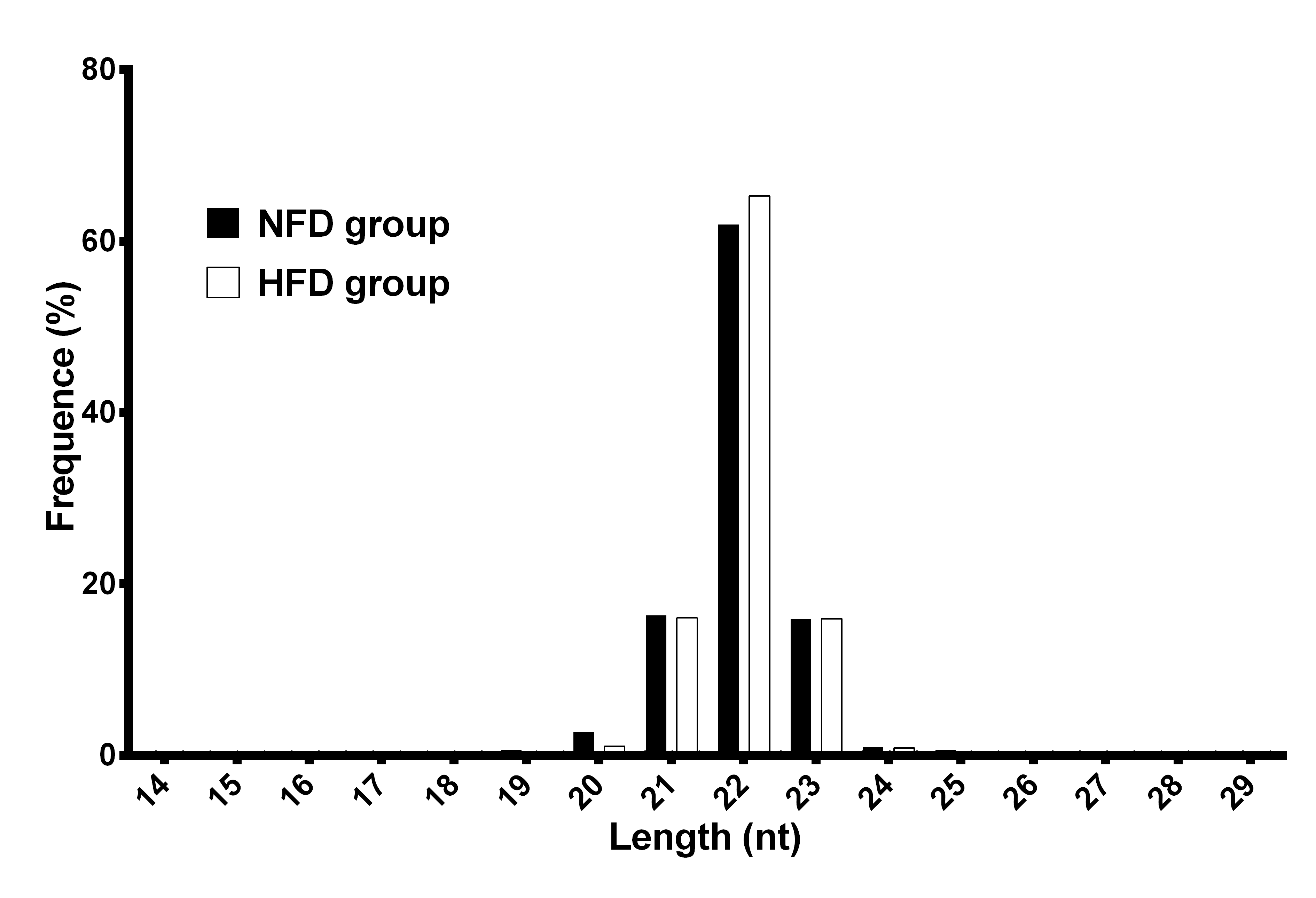

Supplement: Figure S1 — Size distributions of small RNAs identified in the normal-fat diet and high-fat diet libraries. (TIF) [file pone.0096132.s001.tif]

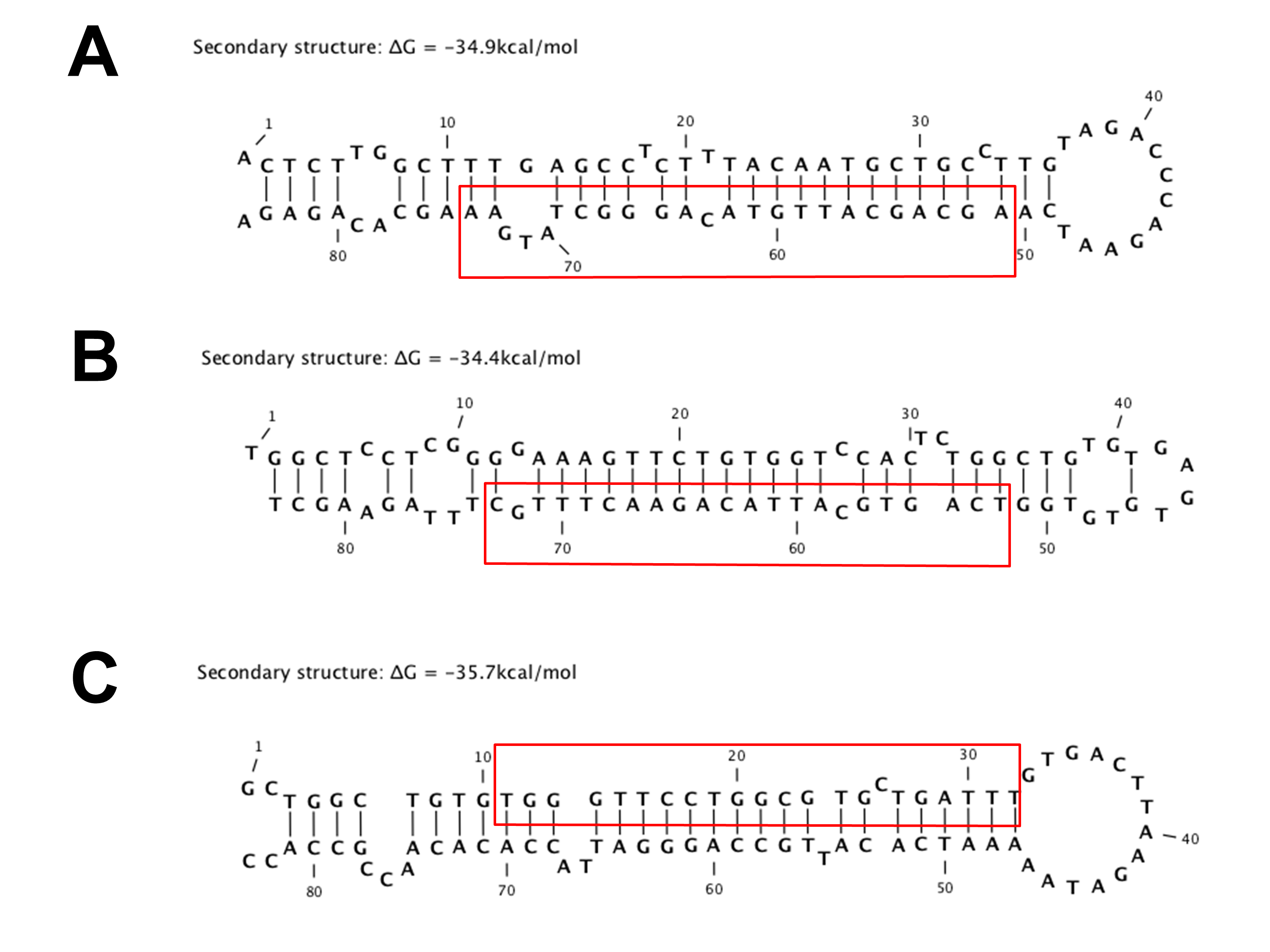

Supplement: Figure S2 — Sequences and structures of the three novel miRNAs identified in the normal-fat diet and high-fat diet groups. Precursor sequences, predicted stem-loop structures and minimum free energies (△G in kcal/mol) of novel_mir-2 (A), novel_mir-4 (B), and novel_mir-7 (C). The sequences of the corresponding mature miRNAs are indicated by red rectangles. (TIF) [file pone.0096132.s002.tif]

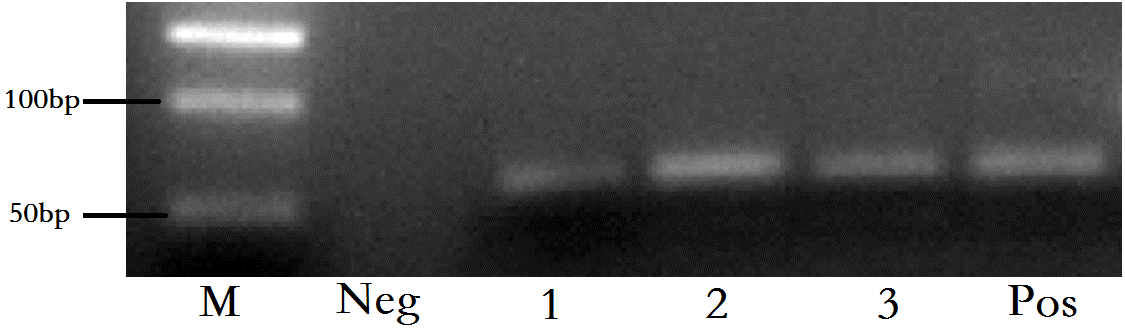

Supplement: Figure S3 — Stem-loop reverse transcriptase-polymerase chain reaction confirmation of the expression of three novel miRNAs in blunt snout bream. M, DNA marker; Neg, negative control (no DNA/RNA); lane 1, novel_mir-2; lane 2, novel_mir-4; lane 3, novel_mir-7; Pos, positive control (endogenous miR-122). (TIF) [file pone.0096132.s003.tif]
